
JOURNAL INFORMATION
==============================
NLM Title Abbreviation: Tex Med J (Austin)
NLM Journal ID: 21130590R

Texas Medical Journal

ISSN: 0892-8495
Publisher: Biomedical Journal Digitization Project

ARTICLE INFORMATION
==============================
PMCID: PMC9615888
Article version: 1
Subjects: Books and Magazines

Dorland’s Modern Obstetrics

Modern Obstetrics: General and Operative

Print publication date: 1902 Feb
Volume: 17
Issue: 8
First page: 311
Last page: 311
License: This work is free of known copyright restrictions. For more information, please see PMC Back Issue Digitization.
License URL: https://creativecommons.org/publicdomain/mark/1.0/

==============================

### Phillips' Emulsion

50% best NORWAY COD LIVER OIL
minutely sub-divided,
WITH WHEAT PHOSPHATES (Phillips')

Pancreatized, Palatable, Permanent, Miscible in Water, Milk, Wine, etc.

### Phillips' Milk of Magnesia

Mg H2 O2 (FLUID.)
"THE PERFECT ANTACID."

for correcting Hyperacid conditions—local or systemic.
Vehicle for Salicylates, Iodides, Balsams, etc.

### Phillips' Phospho-Muriate of Quinine, COMP.

TONIC AND RECONSTRUCTIVE.

WHEAT PHOSPHATES, WITH MURIATE OF QUININE AND STRYCHNINE.

PHILLIPS' WHEAT PHOSPHATES (ACID).
PHILLIPS' SYRUP OF WHEAT PHOSPHATES.
PHILLIPS' DIGESTIBLE COCOA.

THE CHAS. H. PHILLIPS CHEMICAL CO., NEW YORK

Dorland's Modern Obstetrics.—Modern Obstetrics: General and Operative.—W. A. Newman Dorland, A. M., M. D., Assistant Demonstrator of Obstetrics, University of Pennsylvania; Associate in Gynecology, Philadelphia Polyclinic. Second edition, rewritten and greatly enlarged. Handsome octavo, 797 pages, with 201 illustrations. Philadelphia and London: W. B. Saunders & Co. 1901. Cloth, $4.00, net.

This volume contains a complete and systematic presentation of present-day obstetrics as practiced by the best obstetricians of this country and Europe.

The text is so paragraphed, italicised and numbered that it is usually easy of reference. It has been liberally provided with diagramatic tables and well executed illustrations, both of which enhance considerably its value.

In his revision of the original text for the present edition, Dr. Dorland has rewritten and enlarged many parts and has added new chapters in the surgical treatment of puerperal sepsis, infant mortality, placental traumatism of disease, serum therapy of puerperal sepsis and the roll of the liver in the production of puerperal eclampsia. R.
